# Physical Activity and All-cause Mortality in Japan:  The Jichi Medical School (JMS) Cohort Study

**DOI:** 10.2188/jea.JE20080043

**Published:** 2009-01-30

**Authors:** Shinya Hayasaka, Yosuke Shibata, Shizukiyo Ishikawa, Kazunori Kayaba, Tadao Gotoh, Tatsuya Noda, Chiyoe Murata, Tomoyo Yamada, Yasuaki Goto, Yosikazu Nakamura, Toshiyuki Ojima

**Affiliations:** 1 Department of Community Health and Preventive Medicine, Hamamatsu University School of Medicine, Hamamatsu, Shizuoka, Japan; 2 Division of Community and Family Medicine, Center for Community Medicine, Jichi Medical University, Shimotsuke, Tochigi, Japan; 3 School of Health and Social Services, Saitama Prefectural University, Moroyama, Saitama, Japan; 4 Japan Health and Research Institute, Tokyo, Japan; 5 Department of Public Health, Jichi Medical University, Shimotsuke, Tochigi, Japan

**Keywords:** all-cause mortality, cohort study, Japan, physical activity

## Abstract

**Background:**

In April 2008, a new health check-up and health guidance system was introduced by the Japanese Government to promote increased physical activity. However, few studies have documented the health benefits of physical activity in Asian populations. We examined the association between all-cause mortality and level of physical activity in a Japanese multicommunity population-based study.

**Methods:**

The Jichi Medical School Cohort Study is a multicommunity, population-based study based in 12 districts in Japan. Baseline data from 4222 men and 6609 women (mean age, 54.8 and 55.0 years, respectively) were collected between April 1992 and July 1995. The participants were followed for a mean duration of 11.9 years. To determine the association between all-cause mortality and level of physical activity, crude mortality rates per 1000 person-years and hazard ratios (HRs) with 95% confidence intervals (CI) were determined using the Cox proportional hazards model. Physical activity was categorized by using physical activity index (PAI) quartiles. The lowest (first) PAI quartile was defined as the HR reference.

**Results:**

In men, the lowest mortality rate was observed in the third quartile, with 95 deaths and a crude mortality rate of 7.6; the age- and area-adjusted HR was 0.59 (95% CI, 0.45–0.76), and the mortality curve had a reverse J shape. In women, the lowest mortality rate was observed in the highest PAI quartile, with 69 deaths and a crude mortality rate of 3.5; the HR was 0.81 (95% CI, 0.58–1.12).

**Conclusion:**

Our results suggest that increased physical activity lowers the risk for all-cause death in Japanese.

## INTRODUCTION

In April 2008, a new health check-up and health guidance system entitled, “Health Checkups and Healthcare Advice with Particular Focus on Metabolic Syndrome,” was introduced by the Japanese Government. As part of the system, all people aged 40 to 74 are now requested to undergo a health check-up and health guidance aimed at decreasing the incidence of metabolic syndrome and lifestyle-related diseases.^1^ As part of the guidance provided, emphasis is placed on the importance of increasing physical activity.^1^

In recent years, many epidemiological studies have confirmed the health benefits of physical activity,^2^^–^^6^ noting an association between a high level of physical activity and lower mortality. However, most of these studies have been conducted in Western countries; only a few have been conducted within Asian populations. Furthermore, the few studies that have been conducted in Asia were performed in a particular community or company; only rarely have multiple communities been investigated.

The purpose of this study was to examine the association between all-cause mortality and levels of physical activity in Japan using a multicommunity, population-based approach.

## METHODS

The Jichi Medical School (JMS) Cohort Study is a prospective, population-based study aimed at exploring the risk factors for cerebro-cardiovascular disease in 12 communities in Japan.^7^^–^^10^ Enrollment into the JMS Cohort Study and baseline data collection were performed between April 1992 and November 1993. Details regarding the JMS Cohort Study design and additional descriptive data are available elsewhere.^7^^–^^10^ The response rate for the check-up for all communities was 65%.^8^^,^^10^ A total of 12,490 participants (4911 men and 7579 women) participated. Of these, we were able to follow 12,393 participants, among whom physical activity data were available for 11,634 (4549 men and 7085 women). We excluded 803 participants with a history of stroke, cardiovascular disease, or carcinoma. Ultimately, data from 10,831 participants (4222 men and 6609 women) were analyzed in the present study.

To ensure uniformity in data collection, we established a central committee comprising the chief medical officers of all the participating areas. This committee developed a detailed manual for data collection. Smoking habits were assessed using a questionnaire developed for the study.^7^ Smokers were defined as participants who smoked at the time of the study. Body mass index was defined as weight (kg) divided by the square of body height (m). Systolic blood pressure was measured using an automated sphygmomanometer (BP203RV-II, Nippon Colin, Komaki, Japan) on the right arm of seated participants, after at least 5 minutes seated rest. Serum total cholesterol was measured by an enzymatic method (Wako, Osaka, Japan; interassay coefficient of variation (CV), 1.5%).

Physical activity was assessed according to the criteria used in The Framingham Study. A questionnaire^11^ was administered in an interview conducted by a trained reviewer. Information obtained included average sleeping time, working hours, and the kinds of activities conducted during a typical workday and during leisure time in a normal weekday. We classified activities into 5 groups according to level of exertion. Each level of exertion was assigned a coefficient based on the Framingham Study’s physical activity index weighting factors.^11^ The coefficients and time spent on an activity were then multiplied. We then summed the multiplied values to produce the Physical Activity Index (PAI) over 24 hours. For example, “a person who sleeps continually would receive a score of 24. An office worker with no outside exercise could have a score of 27 (8 hours at a basal level, 12 hours at a sedentary level, and 4 hours at a slight level of activity). A laborer who is involved in heavy activity in his job could have a score of 42 (8 hours at a basal level, 8 hours at a sedentary level, 2 hours at a slight level, 3 hours at a moderate level, and 3 hours at a heavy level of activity)”.^11^

Information on deaths was collected using data from death certificates and the national vital statistics database with the permission of the Agency of General Affairs and the Ministry of Health, Labour and Welfare, Japan. In addition, municipal governments obtained information annually on participants who moved to other areas. Written informed consent was obtained individually from all participants at the mass screening examination. The Institutional Review Board of JMS was responsible for ethical review of this research and approved this study.

### Statistical analyses

We categorized participants into 4 groups according to their PAI quartile. First, potential confounders by PAI quartile were expressed as means plus standard deviation (SD). *P* values were calculated using one-way analysis of variance for variables. Smoking status was tested using the chi-square test. Next, crude mortality rates were calculated per 1000 person-years and categorized by sex and PAI. Finally, the Cox proportional hazards model was used to calculate hazard ratios (HRs) and 95% confidence intervals (95% CI) for all-cause mortality adjusted for age and area (model 1), and adjusted for age, area, smoking status, body mass index, systolic blood pressure, and total cholesterol (model 2), in men and women. All statistical analyses were performed using the Statistical Package for Social Science® (SPSS) for Windows (SPSS Japan Inc., version 15.0, Tokyo, Japan).

## RESULTS

Participants were followed for an average of 11.9 years from the time of baseline measurement until either death due to any cause, incident cardiovascular disease, relocation to another area, or the end of the study; follow-up was completed for 99% of the cohort. Participant age at baseline was 54.8 ± 12.0 years (mean ± SD) for men and 55.0 ± 11.3 for women. During the study period there were 834 deaths: 503 men and 331 women.

Tables 1 and 2 show the mean values and SD for potential confounders, by PAI quartile, in men and women, respectively. There were significant differences between quartiles in many potential confounders; however, the mean values for these potential confounders and for smoker rates did not vary considerably across physical activity groups.

**Table 1.** Potential confounders categorized by PAI (men)

		Quartile 1 (≤28.8)			Quartile 2 (28.9–34.3)			Quartile 3 (34.4–38.4)			Quartile 4 (≥38.5)			
Factors		n	Mean	SD	n	Mean	SD	n	Mean	SD	n	Mean	SD	*P*
Age	(year)	1046	54.8	13.0	1046	57.0	12.0	1045	54.3	11.4	1059	53.1	11.1	<0.001
Body mass index	(kg/m^2^)	1028	23.2	2.9	1032	23.0	2.9	1014	22.8	2.8	1041	22.8	2.8	0.006
Systolic blood pressure	(mm Hg)	1037	131.2	20.4	1040	132.1	20.4	1027	131.4	20.1	1045	129.4	20.6	0.019
Total cholesterol	(mg/dl)	1037	187.8	35.6	1038	185.5	34.6	1029	183.4	33.1	1048	182.9	33.8	0.004
Smoker	n (%)	545	(52.4%)		505	(48.4%)		554	(53.2%)		540	(51.2%)		<0.001

*P* values were calculated using the chi-square test for smokers and one-way analysis of variance for others.

SD: standard deviation

**Table 2.** Potential confounders categorized by PAI (women)

		Quartile 1 (≤28.0)			Quartile 2 (28.1–30.2)			Quartile 3 (30.3–33.9)			Quartile 4 (≥34.0)			
Factors		n	Mean	SD	n	Mean	SD	n	Mean	SD	n	Mean	SD	*P*
Age	(year)	1633	53.9	12.8	1630	54.7	11.7	1652	55.8	10.7	1642	55.8	9.6	<0.001
Body mass index	(kg/m^2^)	1604	23.0	3.3	1591	23.2	3.2	1623	23.1	3.2	1614	23.1	3.1	0.509
Systolic blood pressure	(mm Hg)	1622	126.9	21.3	1614	127.9	22.0	1638	128.4	20.6	1618	128.3	20.2	0.148
Total cholesterol	(mg/dl)	1620	197.1	35.7	1612	197.4	35.5	1644	198.6	34.5	1628	193.7	33.7	<0.001
Smoker	n (%)	114	(7.1%)		107	(6.7%)		81	(5.0%)		63	(3.9%)		<0.001

*P* values were calculated using the chi-square test for smokers and one-way analysis of variance for others.

SD: standard deviation

Table 3 shows per 1000 person-years, categorized by sex and PAI. In men, this rate was lowest in the third quartile (Quartile 3), with 95 deaths and a crude mortality rate of 7.6. It was highest in the lowest PAI quartile (Quartile 1), with 171 deaths and a crude mortality rate of 14.4. In women, the crude mortality rate was lowest in the highest PAI quartile (Quartile 4), with 69 deaths and a crude mortality rate of 3.5, and highest in Quartile 1, with 99 deaths and a crude mortality rate of 5.2.

**Table 3.** Number of all-cause deaths and crude mortality rates per 1000 person-years categorized by PAI

	Men				Women		
		Death				Death	
			
PAI category	Person-years	n	Rate	PAI category	Person-years	n	Rate
Total	49,124	503	10.2	Total	77,693	331	4.3
Quartile 1 (≤28.8)	11,844	171	14.4	Quartile 1 (≤28.0)	18,891	99	5.2
Quartile 2 (28.9–34.3)	12,220	130	10.6	Quartile 2 (28.1–30.2)	19,197	76	4.0
Quartile 3 (34.4–38.4)	12,472	95	7.6	Quartile 3 (30.3–33.9)	19,634	87	4.4
Quartile 4 (≥38.5)	12,588	107	8.5	Quartile 4 (≥34.0)	19,971	69	3.5

Table 4 shows the HRs and 95% CIs for model 1 and 2 calculated using the Cox proportional hazards model for groups categorized by PAI; the lowest PAI group was used as the reference. In men, the HRs for the third quartile (Quartile 3) were the lowest: 0.59 (95% CI, 0.45–0.76) for both model 1 and 2; the mortality curve had a reverse J shape. In women, the HRs for the second PAI quartile (Quartile 2) were the lowest: 0.77 (0.57–1.05) for model 1 and 0.79 (0.58–1.08) for model 2. In both sexes, the HRs of higher PAI groups tended to be lower than those of the lowest PAI (reference) group.

**Table 4.** Adjusted hazard ratios for death from any cause

	Men			
	Model 1*		Model 2^†^	
PAI Quartile	HR	95% CI	HR	95% CI
		
Quartile 1 (≤28.8)	1.00		1.00	
Quartile 2 (28.9–34.3)	0.66	0.52 – 0.83	0.69	0.55 – 0.88
Quartile 3 (34.4–38.4)	0.59	0.45 – 0.76	0.59	0.45 – 0.76
Quartile 4 (≥38.5)	0.76	0.59 – 0.98	0.75	0.55 – 1.04

	Women			
	Model 1*		Model 2^†^	
	HR	95% CI	HR	95% CI
		
Quartile 1 (≤28.0)	1.00		1.00	
Quartile 2 (28.1–30.2)	0.77	0.57 – 1.05	0.79	0.58 – 1.08
Quartile 3 (30.3–33.9)	0.89	0.66 – 1.20	0.90	0.66 – 1.22
Quartile 4 (≥34.0)	0.81	0.58 – 1.12	0.83	0.59 – 1.16

Model 1*: adjusted for age and area

Model 2^†^: adjusted for age, area, smoking status, body mass index, systolic blood pressure, and total cholesterol

HR: Hazard ratio; 95% CI: 95% confidence interval

## DISCUSSION

Our multicommunity, population-based study found that, among both men and women, groups with a higher PAI had a lower risk of all-cause death than groups with a lower PAI. This was demonstrated by the fact that the groups with a higher PAI had lower crude mortality rates and lower HRs than groups with a lower PAI (Tables 3 and 4).

Many previous studies have examined the health benefits of physical activity. Demonstrated benefits include control of bodyweight; improvement of hyperlipidemia, hypertension, osteoporosis, sleep disorders, and anxiety; and prevention of diseases associated with aging.^2^ Previous epidemiological studies have also revealed that physical activity is associated with lower mortality.^2^^–^^6^ Our findings are compatible with these previous studies. In addition, many previous studies have shown a dose-response relationship between physical activity and all-cause mortality, and some of the reported data were compatible with a J-shaped, reverse J-shaped, or U-shaped mortality curve.^3^^,^^4^ Our study showed a reverse J-shaped curve in men, suggesting that moderate physical activity is indeed beneficial. The group with the highest PAI had a higher death rate than groups in the second and third quartiles, but a lower death rate than the group with the lowest PAI. In a comparison of highly active and moderately active individuals, Kiely DK et al observed that vigorous physical activity yielded no additional protective effect against stroke.^12^ Others have suggested that the risk for ischemic heart disease increases with physical activity levels beyond moderate.^13^^,^^14^ A previous cohort study in a Japanese provincial city also revealed a U-shaped relationship between physical activity index and incidence of all strokes.^15^^,^^16^ They concluded that individuals performing heavy physical labor were at higher risk for cardiovascular disease.

In women, an inverse relationship between physical activity and the all-cause death rate was observed; however, this finding was not statistically significant. This lack of statistical significance may have been due to the fact that there were fewer deaths among women than men. In addition, the majority of women were classified as housewives, and it is possible that the level of physical activity associated with housework was underestimated.^12^ Using data from 38 studies to estimate the average effect of physical activity and fitness on mortality, Oguma et al calculated that the median relative risk for death among highly active individuals was 0.66.^4^ Our HR results are in conformity with this previous result.

In order to exclude the effects of participants with latent diseases, we also calculated HRs for all-cause mortality after excluding deaths that occurred during the first 2 years of follow-up. However, the trends for HRs calculated with and without this exclusion were similar.

Our study does possess some limitations. First, the measure of physical activity used was based on participant recall, and thus, there is likely to have been considerable imprecision and some misclassification of participants. However, this blunt classification is likely to result in an underestimation of the association between physical activity and mortality.^11^ Second, we obtained information about physical activity only once, at baseline. Katzmarzyk et al reported that less physically active people who increase their physical activity after initial data collection have a lower mortality rate than people who remain less active.^17^ However, this second limitation is also likely to have resulted in an underestimation of the association between physical activity and mortality.

In conclusion, our multicommunity, population-based study found that a higher level of physical activity is associated with a lower risk for all-cause death in men and women. These findings may encourage people to increase their level of physical activity in accordance with the aims of the new Japanese health check-up system.
